# Uterine prolapse and its impact on quality of life in the Jhaukhel–Duwakot Health Demographic Surveillance Site, Bhaktapur, Nepal

**DOI:** 10.3402/gha.v8.28771

**Published:** 2015-08-10

**Authors:** Binjwala Shrestha, Sharad Onta, Bishnu Choulagai, Rajan Paudel, Max Petzold, Alexandra Krettek

**Affiliations:** 1 Department of Community Medicine and Public Health, Maharajgunj Medical Campus, Institute of Medicine, Tribhuvan University, Kathmandu, Nepal; 2 Department of Internal Medicine and Clinical Nutrition, Institute of Medicine, Sahlgrenska Academy at University of Gothenburg, Gothenburg, Sweden; 3 Health Metrics, Institute of Medicine, University of Gothenburg, Gothenburg, Sweden; 4 School of Public Health, Faculty of Health Sciences, University of the Witwatersrand, Johannesburg, South Africa; 5 Department of Biomedicine and Public Health, School of Health and Education, University of Skövde, Skövde, Sweden; 6 Department of Community Medicine, Faculty of Health Sciences, The Arctic University of Norway, Tromsø, Norway

**Keywords:** uterine prolapse, quality of life, Health Demographic Surveillance Site, Nepal

## Abstract

**Background:**

Uterine prolapse (UP) is a reproductive health problem and public health issue in low-income countries including Nepal.

**Objective:**

We aimed to identify the contributing factors and stages of UP and its impact on quality of life in the Jhaukhel–Duwakot Health Demographic Surveillance Site of Bhaktapur, Nepal.

**Design:**

Our three-phase study used descriptive cross-sectional analysis to assess quality of life and stages of UP and case–control analysis to identify contributing factors. First, a household survey explored the prevalence of self-reported UP (Phase 1). Second, we used a standardized tool in a 5-day screening camp to determine quality of life among UP-affected women (Phase 2). Finally, a 1-month community survey traced self-reported cases from Phase 1 (Phase 3). To validate UP diagnoses, we reviewed participants’ clinical records, and we used screening camp records to trace women without UP.

**Results:**

Among 48 affected women in Phase 1, 32 had Stage II UP and 16 had either Stage I or Stage III UP. Compared with Stage I women (4.62%), almost all women with Stage III UP reported reduced quality of life. Decreased quality of life correlated significantly with Stages I–III. Self-reported UP prevalence (8.7%) included all treated and non-treated cases. In Phase 3, 277 of 402 respondents reported being affected by UP and 125 were unaffected. The odds of having UP were threefold higher among illiterate women compared with literate women (OR=3.02, 95% CI 1.76–5.17), 50% lower among women from nuclear families compared with extended families (OR=0.56, 95% CI 0.35–0.90) and lower among women with 1–2 parity compared to >5 parity (OR=0.33, 95% CI 0.14–0.75).

**Conclusions:**

The stages of UP correlated with quality of life resulting from varied perceptions regarding physical health, emotional stress, and social limitation. Parity, education, age, and family type associated with UP. Our results suggest the importance of developing policies and programs that are focused on early health care for UP. Through family planning and health education programs targeting women, as well as women empowerment programs for prevention of UP, it will be possible to restore quality of life related to UP.

Uterine prolapse (UP) occurs when weakness in normally supportive tissues cause the uterus to descend, with or without the urinary bladder and bowel, into the vagina (1). Common symptoms include pelvic pressure, discomfort, visible bulging, and sexual impairment (2). Clinically, UP symptoms are organized into four groups according to presentation: vaginal, urinary, bowel, and sexual (3).

Clinicians grade quality of life according to perceived symptoms and experiences in daily life (4) and determine severity according to the degree, or stage, of prolapse (5). Because women usually fail to recognize the early symptoms of UP, doctors often identify Stage I during clinical examination. In Stage II, women may experience symptoms but frequently do not seek help. Symptoms become more severe as the uterus drops further into the vaginal canal (Stage III). In Stage IV, the uterus protrudes from the vagina, requiring emergency care (6). Depending on stage, UP can greatly impair women's ability to work, which is particularly significant in societies that link women's value with their ability to work (7). Our recent study in the Dhading district of Nepal reported similar experiences (8).

Risk factors of UP include age, parity, and predisposing factors such as obstetric conditions resulting from excessive stretching and tearing, multiple deliveries (9–11), vaginal delivery, and high body mass index (12). In Nepal, key risk factors include extensive physical work during pregnancy and immediately after delivery, as well as the use of unskilled birth attendants (13).

As determined by a systematic review of UP studies from low- and lower middle-income countries among approximately 83,000 women, UP prevalence is 19.7% (range=3.4–56.4%) (9). A study in eight districts in Nepal reported the national UP prevalence as 10% (14), and prevalence in a prospective study among 1,337 women of reproductive age in the Bhaktapur district was 7.55% (15). Estimates by the United Nations Population Fund suggest that about 600,000 Nepalese women require immediate health care for UP (16).

Due to the high prevalence of UP and possible risk factors, the Government of Nepal has developed preventive strategies and curative health care policies. The National Safe Motherhood Program (Aama program) and a family planning program under essential health care package, target primary prevention (17). Policy documents for secondary prevention include an operational guideline for UP management and a protocol for surgical treatment. The guideline mainly highlights quality of care and includes a policy that provides an incentives package for surgical treatment for UP targeting low-income women (18). The government also conducts UP screening camps and recommends annual targets for surgical treatment for UP. Although surgical treatment significantly improves quality of life, it is not offered to all UP-affected women. Therefore, it is better to focus on preventive programs and early management of symptoms (19).

This study aimed to increase knowledge of UP by identifying contributing factors and assessing quality of life for UP Stages I–III in the peri-urban Jhaukhel–Duwakot Health Demographic Surveillance Site (JD-HDSS) outside Kathmandu. Our results will help develop future strategies for prevention and timely care of UP, particularly regarding possible risk factors.

## Methods

### Study design

We used descriptive cross-sectional analysis to assess quality of life and stages of UP and case–control analysis to identify contributing factors.

### Setting and participants

**Fig. 1:**
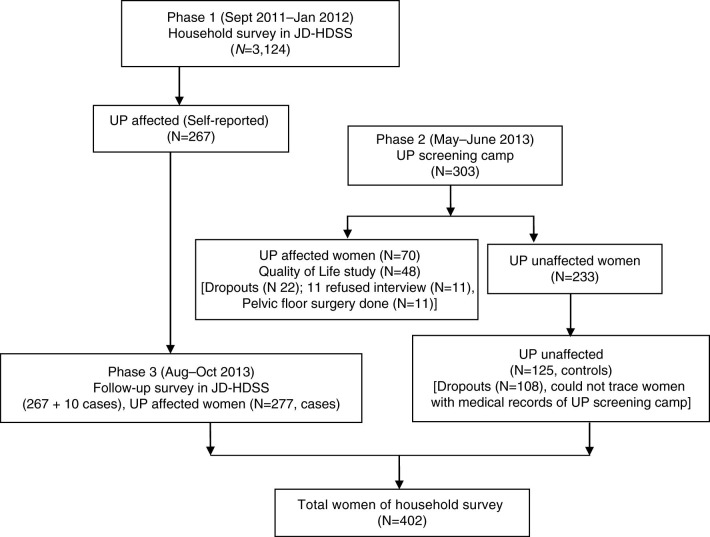
Flow diagram of the three study phases. During Phase 1, we conducted a household survey to identify the prevalence of self-reported uterine prolapse (UP) in the Jhaukhel–Duwakot Health Demographic Surveillance Site (JD-HDSS), Bhaktapur, Nepal. In Phase 2, we organized a 5-day UP screening camp to study how quality of life relates to the stages of UP. Phase 3 included a follow-up community survey as a case–control study to identify factors that associate with UP.

Jhaukhel and Duwakot are village development committees (VDCs) in the Bhaktapur district, 13 km outside Kathmandu. Our group established JD-HDSS in 2010 (20). The current study was conducted in three phases (Fig. 1). During Phase 1, we identified self-reported UP as part of our previous study on the assessment of prevalence and knowledge of UP (21). In Phase 2, we organized a 5-day UP screening camp in JD-HDSS to confirm UP diagnoses, assess quality of life in relation to UP, and to provide UP treatment as needed. Phase 3 was a follow-up community survey, as most women who had self-reported UP did not attended the UP screening camp (Phase 2) despite household invitations by female community volunteers. We employed a case control study design. Based on records from Phase 1, we identified UP-cases from the UP screening camp in Phase 2. Women who were diagnosed as being free of UP were designated as control group.

#### Phase 1 (September 2011–January 2012)

We surveyed 3,124 women of reproductive age and elderly in JD-HDSS and identified 267 women with self-reported UP among women in both reproductive and elderly age groups.

#### Phase 2 (May–June 2013)

We organized a 5-day UP screening camp in JD-HDSS for the UP-affected women identified in Phase 1. We invited women by mobilizing 18 local female community health volunteers of all wards of Jhaukhel and Duwakot VDC. They were oriented about the names and addresses of women who had self-reported UP during the household survey in Phase I. The female community health volunteers then invited these women to attend the screening camp and informed them about the services that would be provided (i.e. clinical checkup, medicine, and referral to hospital for free UP treatment).

In the UP screening camp in Phase 3, there were 303 attendees, 70 were diagnosed with UP and 233 were unaffected by UP. Eleven UP-affected women had undergone hysterectomy for UP and were excluded from the study. The remaining women (*N*=59) had Stage I, II, or III UP. We scored diagnoses according to participants’ answers to questionnaire for pelvic organ prolapse (22), administered by a team of gynecologists from Kathmandu Medical College and Nepal Medical College. Among 59 only 48 women diagnosed with UP participated in a quality of life interview and 11 refused to participate. The reasons for refusing participation were household work load and feeling shame to share personal sexual and reproductive experiences.

#### Phase 3 (August–October 2013)

Following the screening camp (Phase 2), we conducted a community study to trace women previously identified as UP-affected and UP-unaffected women in JD-HDSS (*N*=402), including 267 who participated in the Phase 1 household survey and an additional 10 UP-affected women who were detected in Phase 3 but failed to report to the Phase 1. We could trace 125 UP-unaffected women (controls) of the 233 identified at the UP screening camp during Phase 2.

### Data collection

#### Phase 1

Trained enumerators conducted interviews with women residing in all JD-HDSS households. General questions probed history of UP and care practices.

#### Phase 2

To assess quality of life, one trained female nurse interviewed screening camp participants using a standardized 27-item questionnaire representing nine domains: general health perception; impact of prolapse (vaginal, urinary, bowel, and sexual discomfort); daily household roles; physical and social limitations; personal relationships; emotional problems; sleep energy disturbances; and measurements of symptom severity (4). All questions were translated into Nepali language and pre-tested among women undergoing gynecological examination at Kathmandu Medical College Teaching Hospital. Clinical diagnosis of UP was determined using the standard clinical tool of pelvic organ prolapse questionnaire (POPQ) (22).

#### Phase 3

We trained four local female researchers to conduct interviews with women affected and unaffected by UP. We used the snowballing sampling method to identify participants (23). Before each interview, we validated UP diagnoses using clinical reports such as prescriptions and discharge slips.

Interviews with controls excluded UP diagnosis using records from the screening camp. One author (BS) conducted orientation and supervised the data collection process. We pretested the questionnaire among women in the same VDC and did not include them in the study.

### Data analysis

We used EPIData Manager, version 1 (EpiData Association, Odense, Denmark) for data entry and the Statistical Package for Social Sciences (SPSS) and version 17.0 (SPSS Inc., Chicago, IL, USA) for statistical analysis. We used descriptive statistics to describe participants’ socioeconomic characteristics. In addition, we computed the proportion of socioeconomic characteristics for interviewees who participated in both the screening camp and the household survey.

#### Quality of life

First, we determined the proportion of diagnosis type and stage of UP. The main questions to assess quality of life were grouped into nine domains such as: 1) general health perception due to UP in daily life (physical symptoms and back pain); 2) impact of UP in quality of life due to physical discomfort (vaginal, urinary, bowel, and sexual symptoms); 3) effect on daily household roles (outdoor and physical work); 4) impact on physical activities (walking, sitting, sleeping, and standing); 5) impact on emotional status (feeling of loneliness, sadness, and self-blame); 6) impact on sleep energy (bad dreams and tiredness); 7) impact on social activities (social work, meeting friends, and family life); 8) impact on personal life (spousal relationship and effects in sexual relationship); and 9) severe measures due to UP (need to use pad or other protective material, pulling up uterus manually). To score quality of life, we characterized all answers to the questionnaire and the related probing questions as individual variables. Next, we computed individual variables in the nine domains of quality of life, scoring 0 for ‘no effect’ or ‘some effect’ and 1 for ‘little bit effect’ and ‘bad effect’ (4). Finally, we used descriptive statistics and analysis of variance (ANOVA) to determine in all nine domains of total quality of life among three groups of women diagnosed with UP (Stages I–III).

#### Contributing factors

After describing the proportion of socioeconomic characteristics of women affected and unaffected by UP, we used data from the household survey to compute the associated factors with bivariate and multivariate logistic regression analysis. To check collinearity prior to regression analysis, we calculated the variance inflation factor of all variables. We detected no problem of collinearity among the independent variables. Our multiple logistic regression analyses included all independent variables that were significant at 15% (24) in the simple regression analyses. Statistical significance was set at *p*≤0.05.

### Ethical considerations

Before conducting the interviews, we explained the objective of the study to all participants. Because UP is considered as a private matter in Nepalese society, we ensured respondents’ autonomy and confidentiality. All interviewees provided verbal consent. The Nepal Health Research Council granted ethical approval for this study (Reg. no. 56/2012).

## Results

### Phase 1: Household survey

#### Participant characteristics

During the household survey, 267 (8.8%) women reported UP problems from 3,124 households in Duwakot and Jhaukhel (62.5 and 37.8%, respectively) (self-reported UP prevalence, treated and untreated). Mean age of participants was 45 ±13.1 (SD) years. Caste/ethnic groups included Brahmin/Chhetri (64.4%), Newar (25.5%), Janajati (5.6%), and Dalit (4.5%).

### Phase 2: UP screening camp

#### Participant characteristics

A total of 48 women among the camp attendees (*N*=70 UP diagnosed) participated in Phase 2. Among interviewees, 73.9% were from Jhaukhel and 26.1% from Duwakot. Most participants were Newar (39.1%), followed by Chhetri (34.8%), Brahmin (21.7%), and Dalit (4.3%). Mean age was 50.1±10.9 (SD) years, and mean age at marriage was 18.0±1.9 (SD) years. Participants had their first pregnancy at 21.0±3.2 (SD) years, and each woman had 4.3±2.2 (SD) children. Regarding educational background, 65.2% were illiterate. Men were the decision makers in most families (73.9%). Major occupations included business and service (34.8%) and agriculture (17.4%). Most women (56.5%) lived in extended families (Table 1). The mean duration of UP suffering was 4.63±5.5 (SD) years.

**Table 1:** Distribution of socioeconomic characteristics of participants in the uterine prolapse screening camp in the Jhaukhel–Duwakot Health Demographic Surveillance Site, Bhaktapur, Nepal (Phase 2)

Variable	Participants in screening camp diagnosed as UP N (*N*=48)	%
VDC		
Jhaukhel	35	72.9
Duwakot	13	27.1
Caste/ethnic group		
Brahmin	11	22.9
Chhetri	16	33.3
Newar	19	39.6
Janajati	0	0.0
Dalit	2	4.2
Dalit and non-Dalit		
Non-Dalit^a^	46	95.8
Dalit^b^	2	4.2
Education status		
Illiterate	32	66.7
Educated	16	33.3
Family type		
Nuclear	21	43.8
Extended	27	56.3
Decision maker in family		
Male	35	73.9
Female	13	26.1
Main source of income		
Agriculture	9	18.8
Business	16	33.3
Service	16	33.3
Labor	7	14.6
Income sufficiency		
Six months	2	4.2
More than 6 months	46	95.8

UP=uterine prolapse; VDC=village development committee.

a Non-Dalit (advantaged) group includes Brahmin, Chhetri, Newar, and Janajati.

b Dalit=disadvantaged group (25).

#### Stages of UP and quality of life perceived by screening camp attendees

Among 48 interviewees, two thirds with Stage II UP and one third with Stage I or Stage II UP (Table 2). Table 3 shows total quality of life scores for nine domains in Stages I–III. Women with Stage III UP scored highest (100%), and women with Stage I UP scored lowest (4.62%). The different domains of quality of life, including physical or social limitation and severe symptoms (i.e. severe pain and pulling of the uterus and the need for a pad or other supportive material) correlated significantly with all three stages. However, general health perceptions, impact, emotional stress, sleep energy, and personal relationships did not correlate with disease stage.

**Table 2:** Distribution of stages of uterine prolapse and scores of quality of life perceived by screening camp attendees in the Jhaukhel–Duwakot Health Demographic Surveillance Site, Bhaktapur, Nepal (Phase 2)

UP stage	Participants, *N* (%)	Quality of life scores, *N* (%)
Stage I	8 (16.7)	20 (4.62)
Stage II	32 (66.7)	139 (32.17)
Stage III	8 (16.7)	72 (100)
All stages	48 (100)	231 (53.48)

UP=uterine prolapse.

**Table 3:** Association between stages of uterine prolapse and effects on quality of life as perceived by screening camp attendees in the Jhaukhel–Duwakot Health Demographic Surveillance Site, Bhaktapur, Nepal (Phase 2)

		Association (ANOVA) between effects in quality of life in three different stages of uterine prolapse (*N*=48)			
		
Domains of quality of life		Stage I (*N*=8)	Stage II (*N*=32)	Stage III (*N*=8)	*p*
1.	General health perception	5	28	8	0.09
2.	Impact	4	27	8	0.026
3.	Role limitation	0	18	8	0.000
4.	Physical limitation	0	8	8	0.000
5.	Emotional stress	5	24	8	0.190
6.	Sleep energy	2	15	8	0.005
7.	Social limitation	0	6	8	0.000
8.	Personal relationship	4	11	8	0.003
9.	Severe measure	0	2	8	0.000

### Phase 3: Follow-up community survey

#### Participant characteristics

The follow-up community study of both UP-affected and -unaffected participants included 402 women. Among these, 277 women were UP-affected (cases), including 267 identified during Phase 1 and 10 more detected Phase 3. Because we expected to find asymptomatic UP cases among our participants, we selected unaffected women (controls) based on medical records procured during Phase 2. According to records of the UP screening camp (Phase 2), we were able to identify 125 women attending the screening camp who were unaffected by UP. They were included as controls in Phase 3.

Among 402 Phase 3 participants, 48% were from Duwakot and 52% from Jhaukhel. The dominant ethnic group in Jhaukhel was Newar (42%), compared with Chhetri (34%) in Duwakot. Mean age was 43.7±14 (SD) years, and mean age at marriage was 17.7±3.1 (SD) years. Mean age of first pregnancy was 20.±2.6 (SD) years; each woman had 3.4±1.9 (SD) children. Regarding educational background, 47.8% were illiterate, and men were the decision makers in most families (72.9%). Most participants (53.4%) lived in extended families, and the most common occupations were service (44.5%) and agriculture (23.6%) (Table 4). The mean duration of UP suffering was 9.6±10.5 (SD) years.

**Table 4:** Socioeconomic background of participants in the community survey in the Jhaukhel–Duwakot Health Demographic Surveillance Site, Bhaktapur, Nepal (Phase 3)

	Household survey participants	
	
Variable	*N* (*N*=402)	%
VDC		
Jhaukhel	209	52.0
Duwakot	193	48.0
Caste/ethnic group		
Brahmin	55	13.7
Chhetri	149	37.1
Newar	169	42.0
Janajati	14	3.5
Dalit	15	3.7
Dalit and non-Dalit		
Non-Dalit	387	96.3
Dalit	15	3.7
Education status		
Illiterate	192	47.8
Educated	210	52.2
Family type		
Nuclear	187	46.5
Extended	215	53.4
Decision maker in family		
Male	293	72.9
Female	109	27.1
Main source of income		
Agriculture	94	23.6
Business	74	18.4
Service	179	44.5
Labor	55	13.4
Income sufficiency		
Six months	28	7.0
>6 months	374	93.0

VDC=village development committee.

#### Characteristics of participants

Four hundred and two women from Jhaukhel and Duwakot VDC (63.2 and 36.8%, respectively) participated in the case–control study, 277 as cases and 125 as controls. All participants were Hindu. UP occurred more frequently in Dalit women than other castes/ethnic groups (90% vs. <75%, respectively), and illiterate women had more UP problems than literate women (83 and 56%, respectively). Women older than 60 years were more prone to UP than women aged 41–60 years (90% vs. 83%, respectively). Similarly, most UP-affected women (74.6%) were younger than 20 years of age during their first pregnancy; and 87.6% had >5 pregnancies in their lifetime (Table 5).

**Table 5:** Distribution of socioeconomic characteristics of women with and without uterine prolapse in the Jhaukhel–Duwakot Health Demographic Surveillance Site, Bhaktapur, Nepal (Phase 3) *N*=402

	UP-unaffected women (controls)	UP-affected women (case)
	
Variable	*N*=125 (%)	*N*=277 (%)
Caste/ethnic group		
Brahmin	14 (25.5)	41 (74.5)
Chhetri	55 (36.9)	94 (63.1)
Newar	51 (30.2)	118 (69.8)
Janajati	4 (28.6)	10 (71.4)
Dalit	1 (6.7)	14 (93.3)
Dalit and non-Dalit group		
Non-Dalit	124 (32.0)	263 (68.0)
Dalit	1 (6.7)	14 (93.3)
Type of family		
Extended	75 (40.1)	112 (59.9)
Nuclear	50 (23.3)	165 (76.7)
Decision maker of family		
Male	92 (31.4)	201 (68.6)
Female	33 (30.3)	76 (69.7)
Education status		
Educated	92 (43.8)	118 (56.2)
Illiterate	33 (17.2)	159 (82.8)
Age group (years)		
15–25	43 (58.9)	30 (41.1)
26–40	60 (33.7)	118 (66.3)
41–60	17 (17.0)	83 (83.0)
>60	5 (9.8)	46 (90.2)
Age at first pregnancy (years)		
15–19	46 (25.4)	135 (74.6)
>20	77 (35.2)	142 (64.8)
Parity (*N*)		
1–2	62 (37.8)	102 (62.2)
3–4	50 (34.5)	95 (65.5)
>5	11 (12.4)	78 (87.6)

#### Association between UP and socioeconomic characteristics

Table 6 describes the factors associated with UP among women with different socioeconomic characteristics. Bivariate and multivariate logistic regression analysis showed that that UP associates with education level, parity, and family type. The odds of having UP were threefold higher among illiterate women (OR=3.02, 95% CI 1.76–5.17). Similarly, women from nuclear families were 50% less likely to have UP compared with women from extended families (OR=0.56, 95% CI 0.35–0.90), and the odds were lower among women with 3–4 parity compared to >5 parity (OR=0.33, 95% CI 0.14–0.75). However, subsequent multivariate analysis showed no significant association with UP for age at first pregnancy and gender of decision maker in the family or caste/ethnic group (Dalit and non-Dalit).

**Table 6:** Associated factors of uterine prolapse among 402 study participants in the Jhaukhel–Duwakot Health Demographic Surveillance Site, Bhaktapur, Nepal (Phase 3)

	Bivariate		Multivariate	
		
Variables	Odds ratio	95% CI odds ratio	Odds ratio	95% CI odds ratio
Education				
Literate and educated	1		1	
Illiterate	3.75	2.36–5.97	3.02	1.76–5.17
Dalit and non-Dalit groups				
Dalit (disadvantaged)	1		1	
Non-Dalit (advantaged)	0.15	0.02–1.16	0.14	0.01–1.13
Family type				
Extended	1		1	
Nuclear	0.45	0.29–0.69	0.56	0.35–0.90
Decision maker in family				
Male	1		1	
Female	1.07	0.66–1.72	0.87	0.51–1.48
Age at first pregnancy (years)				
>20	1		1	
15–19	1.59	1.03–2.45	1.47	0.88–2.46
Parity (number)				
>5	1		1	
3–4	0.26	0.13–0.47	0.33	0.14–0.75
1–2	0.23	0.11–0.54	0.41	0.17–0.96

CI=confidence interval.

## Discussion

### Stages of UP and quality of life

Although assessing quality of life during Phase 2 was challenging due to participants’ lack of time, we interviewed 48 participants during the 5-day camp. The stages and symptoms of UP determine quality of life because they affect women's ability to lift, sit, stand, and walk, resulting in reproductive and urinary tract infections, abdominal pain, and pain during intercourse (7, 8). In relation to health problems (e.g. painful and difficult mobility, social isolation, emotional stress, work energy, and sleep disturbance), quality of life associated significantly with frequency of UP. Further, health problems encountered in Stage III affect marital relationship, occupation, monthly income, and healthcare-seeking practices (5).

Our quality of life assessment, with the observed variations in quality of life in UP Stages I–III, demonstrates the importance of early diagnosis and care of UP. Women with Stage III UP had lowest quality of life compared to those with Stages I and II. Most participants did not even recognize Stage I symptoms. This suggests that if women have increased knowledge about UP and receive health care at an early stage, UP could be diagnosed earlier. This would indeed be helpful to prevent progression toward Stage III. This highlights the importance of teaching women about the early symptoms of UP and encouraging early access to healthcare services.

### UP and associated factors

Nepalese women view UP as a personal problem and are ashamed to reveal their condition (8). Our study mirrors such sentiments. Despite receiving personal invitations from female community health volunteers, many women did not attend the UP screening camp. Consequently, we conducted a follow-up community survey and identified 10 additional UP-affected women using records of the household survey as well as snowball sampling.

Importantly, comprehensive and effective health care and development strategies can prevent and manage the burden of UP (26). Non-obstetric risk factors of UP include obesity, heavy lifting, and constipation (11). In Nepal, women commonly perform extensive physical labor during pregnancy and immediately after delivery and also experience unsafe delivery practices attended by unskilled birth attendants (8, 13). However, UP is also linked with culture, discriminatory gender norms, and values that lead to insufficient education, inadequate information about UP, and the absence of quality maternal health care. Nepal's patriarchal system renders women's position in society inferior to that of men (27, 28).

### Women's empowerment and risk factors for UP

In Nepal, only 46% of women make decisions about their own health care, major household purchases, and visits to their family and relatives. Education is a main predictor for increasing women's empowerment in all household decisions (29). Only 27% of our participants made decisions regarding such activities and most were illiterate.

The dominant risk factors of UP are parity and increased age (9, 11). Raising the use of contraceptives and addressing the unmet needs of family planning increased women's empowerment (29), indicating an indirect relationship between parity and empowerment. Our results also demonstrate an association between parity and UP, suggesting that effective family-planning programs could prevent UP.

Hindu extended families share household resources with the families of siblings and their children. Mothers-in-law control most household roles and responsibilities of her daughters-in-law (30). Our results show greater risk for UP among women from extended families compared to nuclear families, possibly due the larger workload in extended families. Thus, family structure can help identify possible obstetric and non-obstetric risk factors for UP.

## Conclusions

The stages of UP correlated with women's quality of life resulting from varied perceptions regarding physical health, emotional stress, and social limitation. Parity, education, age, and family type associated with UP. Our results suggest the importance of developing policies and programs that are focused on early health care for UP. Through family planning and health education programs targeting women, as well as women empowerment programs for prevention of UP, it will be possible to restore quality of life related to UP.

## References

[1] Baessler K, Schussler B, Kathryn LB, Moore HK, Norton AP, Stanton LS (2008). Pelvic floor re-education principles and practice.

[2] Kovoor E, Hooper P (2008). Assessment and management of pelvic organ prolapse. Obstet Gynecol Reprod Med.

[3] Doshani A, Teo ECR, Mayne JC, Tincello GD (2007). Uterine prolapse. BMJ.

[4] Digesu AG, Khullar V, Cardozo L, Robinson D, Salvatore S (2005). P-QOL: a validated questionnaire to assess the symptoms and quality of life of women with urogenital prolapse. Int Urogynecol J.

[5] Fritel X, Varnoux N, Zins M, Breart G, Ringa V (2010). Symptomatic pelvic organ prolapse at midlife, quality of life, and risk factors. Obstet Gynecol.

[6] Streicher LF (2013). Uterine prolapse and pelvic relaxation. http://www.mygyne.info/uterineprolapse.htm.

[7] Bonetti TR, Erpelding A, Pathak LR (2004). Listening to “felt needs”: investigating genital prolapse in Western Nepal. Reprod Health Matters.

[8] Shrestha B, Onta S, Choulagai B, Poudyal A, Pahari DP, Uprety A (2014). Women's experiences and health care-seeking practices in relation to uterine prolapse in a hill district of Nepal. BMC Women's Health.

[9] Walker GJ, Gunasekera P (2011). Pelvic organ prolapse and incontinence in developing countries: review of prevalence and risk factors. Int Urogynecol J.

[10] Kim CM, Jeon MJ, Chung DJ, Kim SK, Kim JW, Bai SW (2007). Risk factors for pelvic organ prolapse. Int J Gynecol Obstet.

[11] Miedel A, Tegerstedt G, Mæhle-Schmidt M, Nyrén O, Hammarström M (2009). Non obstetric risk factors for symptomatic pelvic organ prolapse. Obstet Gynecol.

[12] 
Gyhagen M, Bullarbo M, Nielsen TF, Milsoma I (2013). Prevalence and risk factors for pelvic organ prolapse 20 years after childbirth: a national cohort study in singleton primiparae after vaginal or caesarean delivery. BJOG.

[13] Bodner-Adler B, Shrivastava C, Bodner K (2007). Risk factors for uterine prolapse in Nepal. J Int Urogynecol J Pelvic Floor Dysfunct.

[14] Gurung G, Rana A, Amatya A, Bishta KD, Joshi AB, Sayami J (2007). Pelvic organ prolapse in rural Nepalese women of reproductive age groups: what makes it so common. N J Obstet Gynaecol.

[15] Marattha RK, Shah A (2003). Genital prolapse in women of Bhaktapur. Nepal Med College J.

[16] UNFPA, Government of Nepal (2007). Booklet on uterine prolapse.

[17] Lamichhane P, Tiwari S (2012). Progress report on the Aama/4 ANC Demand side Financing Program, strengthening health system – Improving Services.

[18] Ministry of Health and Population (2009). Operational guideline for uterine prolapse management and surgical services.

[19] UNFPA, Nepal (2013). Health related quality of life of women suffering from pelvic organ prolapse before and 9 to 11 months after surgical interventions.

[20] Aryal UR, Vaidya A, Shakya-Vaidya S, Petzold M, Krettek A (2012). Establishing a Health Demographic Surveillance Site in Bhaktapur district, Nepal: initial experiences and findings. BMC Res Notes.

[21] Shrestha B, Onta S, Choulagai B, Shrestha KB, Petzold M, Krettek A (2014). Knowledge, prevalence and treatment practices of uterine prolapse among women of reproductive age in the Jhaukhel-Duwakot Health Demographic Surveillance Site, Bhaktapur, Nepal. J Kathmandu Med Coll.

[22] Chen GD (2007). Updated definition of female pelvic organ prolapse. Incont Pelvic Floor Dysfunct.

[23] Explorable.com (2009). Snowball sampling. https://explorable.com/snowball-sampling.

[24] Bursac Z, Gauss CH, Williams DK, Hosmer DW (2008). Purposeful selection of variables in logistic regression. BMC Source Code Biol Med.

[25] Pandey JP, Dhakal MR, Karki S, Poudel P, Pradhan MS (2013). Maternal and child health in Nepal: the effects of caste, ethnicity, and regional identity: further analysis of the 2011 Nepal Demographic and Health Survey.

[26] Amnesty International (2014) (2014). Unnecessary burden gender discrimination and uterine prolapse in Nepal.

[27] Pradhan S (2007). Unheeded agonies: a study of uterine prolapse prevalence and its causes in Siraha and Saptari Districts, Nepal.

[28] UNICEF Nepal (2006). Situation of children and in Nepal.

[29] Ministry of Health and Population (MoHP) [Nepal], New Era and ICF International Inc (2012). Nepal demographic and health survey 2011.

[30] Valsiner J (2000). Culture and human development.

